# Metabolic Consequences after Urinary Diversion

**DOI:** 10.3389/fped.2014.00015

**Published:** 2014-03-10

**Authors:** Raimund Stein, Peter Rubenwolf

**Affiliations:** ^1^Division of Pediatric Urology, Department of Urology, Mainz University Medical Center, Johannes Gutenberg University, Mainz, Germany

**Keywords:** urinary diversion, metabolic complications, vitamin B_12_, acidosis, diarrhea

## Abstract

Metabolic disturbances are well-known, but sometimes neglected immediate consequences or late sequelae following urinary diversion (UD) using bowel segments. Whereas subclinical disturbances appear to be quite common, clinically relevant metabolic complications, however, are rare. Exclusion of bowel segments for UD results in loss of absorptive surface for its physiological function. Previous studies demonstrated that at least some of the absorptive and secreting properties of the bowel are preserved when exposed to urine. For each bowel segment typical consequences and complications have been reported. The use of ileal and/or colonic segments may result in hyperchloremic metabolic acidosis, which can be prevented if prophylactic treatment with alkali supplementation is started early. The resection of ileal segments may be responsible for malabsorption of vitamin B_12_ and bile acids with subsequent neurological and hematological late sequelae as well as potential worsening of the patient’s bowel habits. Hence, careful patient and procedure selection, meticulous long-term follow-up, and prophylactic treatment of subclinical acidosis is of paramount importance in the prevention of true metabolic complications.

## Metabolic Alterations and Complications

Whenever bowel segments are excluded from the gastrointestinal tract for urinary diversion (UD), the absorptive surface of the respective segment is irreversibly lost for physiological bowel function. Functional bowel loss affects the absorption of nutrients and water from small and large bowel (1–5). Some of the absorptive and secreting properties of bowel are preserved if exposed to urine, as clearly demonstrated by previous studies (3, 5). Typical metabolic consequences and complications have been reported for each bowel segment. They have been demonstrated to occur more frequently in patients having undergone continent UD due to the use of longer intestinal segments compared with shorter segments required for ileal and colonic conduits (2, 5, 6). Metabolic disturbances involve mainly the electrolytes, loss of bile acids, and malabsorption of vitamin B_12_, the extent of which depends on the length and type of bowel segment used, the duration of the storage interval, and the concentration of the urinary constituents the bowel is exposed to, the degree of atrophy of the bowel mucosa, and, importantly, the patient’s renal and liver function. Further factors include patient age, status post irradiation or chemotherapy and primary or secondary diseases, which may promote or give rise to subsequent metabolic complications (3).

### Electrolytes

One of the most common metabolic consequences and complications are electrolyte imbalances. Whereas the majority of electrolytes freely traverse the intestinal segments across the apical surface of intestinal cells (transcellular movement), there is also some electrolyte movement between the cell borders (paracellular transport) (7).

## Gastric Segments

Hypochloremic hypokalemic metabolic alkalosis may occur when gastric segments are used for UD; this has been reported to be life-threatening in some cases (8). The intestinal hormone gastrin seems to play a major role in this syndrome as with higher gastrin levels metabolic alkalosis becomes more severe (9).

## Jejunum

The use of jejunum may entail hyponatremia, hypochloremia, hyperkalemia, azotemia, and acidosis paralleled by excessive loss of sodium chloride and by free water, which in turn can result in dehydration with subsequent hypovolemia and increased renin and aldosterone levels (6, 10). The more proximal and the longer the jejunal segment used, the more clinically relevant these disturbances may present as (11, 12).

## Ileal and Colonic Segments

Exclusion of ileal and colonic bowel segments may result in hyperchloremic metabolic acidosis. During the past 60 years, the underlying pathophysiological mechanisms have been the subject of intensive studies (7). Beside a complex interplay of various factors, ammonium ions (${\mathrm{NH}}_{4}^{+}$) are believed to play a major role. When colonic or ileal segments are exposed to urine, ionized ammonium and chloride (Cl^−^) are reabsorbed by the mucosa (13–15). Mediated by a sodium–hydrogen antiport, ammonium absorption occurs in exchange of sodium (Na^+^) (16). The exchange of ammonium (${\mathrm{NH}}_{4}^{+}$) for a proton (H+) in turn is coupled with the exchange of bicarbonate for chloride (Cl^−^) (6). Furthermore, ionized ammonium may be also absorbed into the blood through potassium (K^+^) channels (17), resulting in potential bicarbonate and potassium losses.

## Acidosis in Relation to the Different Types of Urinary Diversion

In patients having undergone ileal conduit diversion, mild to moderate acidosis can be expected in up to 15%. Up to 10% of patients will require antiacidotic treatment (18–20). In patients with a continent UD, the contact time of urine is markedly longer and the exposed surface of bowel mucosa is much larger. As a consequence, this can lead to a higher incidence of electrolyte disturbances. Therefore, metabolic acidosis has been reported in up to 50% of patients (7, 21). An elevated serum chloride concentration is associated with a decreased base excess (2, 7, 22). Treatment of hyperchloremic acidosis consists of administration of alkalizing agents. Prophylactic alkali substitution should be commenced at a base excess below −2.5 mmol/L, with the aim of avoiding the long-term complications of clinically evident acidosis (23).

### Bone density

Incorporation of ileal and/or colonic segments into the urinary tract can lead to chronic acidosis, which may play a major role in a decrease of bone mineral density following UD. The occurrence of rickets in children has been previously reported, but constitutes an extremely rare complication in practice. Similarly, osteomalacia and osteoporosis may develop in adults. However, the definition of osteoporosis in younger adults is not clearly established. At least three possible mechanisms underlying osteoporosis after UD have been described. Bone carbonate has the potential to buffer chronic acidosis in exchange for hydrogen ions with subsequent release of calcium into the circulation. Calcium is then cleared by the kidneys (24–26). Importantly, renal tubule calcium reabsorption is directly inhibited by sulfate (7, 27), with chronic acidosis being a cause for increased intestinal sulfate absorption. Furthermore, acidosis has been reported to activate osteoclasts resulting in further bone resorption (28). Finally, impaired intestinal absorption of both calcium and vitamin D may additionally develop in response to ileal resection (2).

With all these different pathophysiological pathways in mind, one could hypothesize that a substantial proportion of patients following UD using bowel segments will suffer from decreased bone mineralization. Indeed, there are some early reports of osteomalacia after ureterosigmoidostomy (29–31), ureterosigmoidostomy (32), Kock pouch (33), and segmental ileal ureteric replacement (34). Interestingly, remineralization of the bones was shown to be possible by correction of acidosis in two early reports (35, 36). Both animal and clinical studies have demonstrated, that the metabolic disturbances are not as severe as commonly assumed, and, even more importantly, that they could be prevented with the proviso that prophylactic treatment is initiated early (1, 26, 37–39). In clinical practice, mild chronic acidosis was shown to be preventable, if a base deficit of more than −2.5 mmol/L was corrected early. In these patients, no signs of bone demineralization were observed (40). If osteomalacia occurs, correction of acidosis, dietary supplements with calcium, vitamin D and, in severe cases, bisphosphonates are recommended (35, 36, 41, 42).

## Growth Retardation

In 1992, Mundy and Nurse as well as Wagstaff et al. found delayed linear growth in some children after UD (43, 44). The Baltimore group in the States observed decreased linear growth in patients with bladder exstrophy who had undergone intestinal augmentation, as opposed to those without augmentation (45). In a frequently cited long-term study of 93 patients with status post various treatment modalities for meningomyelocele (colonic conduit *n* = 2, ileal conduit *n* = 28, conservative treatment *n* = 63) it was shown that those with UD had a decreased linear growth; the rate of complications following orthopedic surgery was 17% compared to 3% in the conservative group (46). Recurrent pyelonephritis occurred in 60% of the patients with a conduit as opposed to “only” 21% in the conservative group; likewise, deterioration of the upper urinary tract was observed in 57 and 8%, respectively. All these factors may compromise renal function and thereby decrease the ability of the kidneys to counteract acidosis, which, as already stated above, has a negative impact on bone mineral density. Twenty percent of the patients with a conduit diversion had intermittent metabolic acidosis. At that time, the incidence of complications following orthopedic procedures in patients with meningomyelocele ranged between 16 and 29% (47, 48).

It is of note, however, that conclusions of all of the above retrospective studies are limited by methodological shortcomings. For instance, it has been clearly demonstrated that a reduced bone mineral density is markedly more common in children with myelomeningocele than in others undergoing UD (49). In support of this, another recently published study provided compelling evidence that there is a significant correlation between low-bone density and wheelchair-dependence in children. Moreover, an association between reduced bone density and higher neurological deficit was suggested (50). Gerharz et al. emphasized, that it is worth to take a second look at the linear growth of patients who had undergone enterocystoplasty in childhood. In their study, the initial series by Wagstaff et al. were incorporated (43, 51). Eighty-five percent of patients remained on the same or reached a higher centile after surgery; only 15% were in a lower position, and clinically relevant growth retardation was recognized in only four patients. All these patients underwent a complete endocrinological evaluation demonstrating that enterocystoplasty was not the underlying cause of growth retardation in a single case. Therefore, it seems very unlikely that the post-operative loss of the changes in position on the growth curve is a consequence of the UD. Rather, it seems to be more likely a non-specific phenomenon that should be considered in any clinical population of similar size and age distribution after the same length of time (51).

## Vitamin B_12_

Vitamin B_12_ (cobalamin) cannot be synthesized by mammalians and must be ingested from food. It plays an important role in DNA synthesis and neurological function. The acidic environment in the stomach facilitates the uncoupling of vitamin B_12_ from food. The parietal cells in the stomach secrete intrinsic factor, which binds to vitamin B_12_ in the duodenum. In the ileum, the vitamin B_12_-intrinsic factor complex helps absorb vitamin B_12_ (52). Moreover, the so-called cubilin receptor, which is found in the entire ileum and not only in the terminal ileum, has also a physiological role in the absorption of vitamin B_12_ (53–55). Additionally, there is some evidence of the existence of an alternative system, which is obviously independent of the intrinsic factor or the terminal ileum and about 1% of orally administered vitamin B_12_ is absorbed by an additional, yet unknown, pathway (52, 56, 57). Collectively, current knowledge suggests that the terminal ileum is not the only site of vitamin B_12_ absorption (56–61). A normal Western-style diet contains approximately 5–15 μg of vitamin B_12_ per day; however, the daily requirement amounts to only 1–2 μg. Furthermore, the large hepatic vitamin B_12_ depot in humans prevents symptoms of vitamin B_12_ deficiency over a period of 2–5 years even in the presence of severe malabsorption (62). On the other hand, overt vitamin B_12_ deficiency may result in megaloblastic macrocytic anemia, Hunter’s glossitis, and funicular myelosis, an irreversible degeneration of spinal cord white matter. In most cases, however, vitamin B_12_ deficiency can remain completely asymptomatic (63, 64). In adults, resection of more than 60 cm of ileum increases the risk of development of a vitamin B_12_ deficiency, as shown by some earlier studies (65–69). In children, impaired vitamin B12 absorption was demonstrated following resection of more than 45 cm of ileum demonstrated impaired vitamin B_12_ absorption (70), whereas another study concluded that vitamin B_12_ absorption in children normalizes after ileal resection (71).

Other studies reported low vitamin B_12_ levels in adults in whom ileal segments had been used for UD. This was particularly evident if longer segments of ileum were used (22, 72–81). However, the length of excluded ileum is not the only contributing factor (82): radiation therapy (83, 84), patient age (85–87), as well as administration of omeprazole (88) may all cause malabsorption of vitamin B_12_.

In children, no decrease in vitamin B_12_ levels was observed following bladder augmentation and substitution using ileal segments inmost cases after a follow-up period of up to 8 years (89). By contrast, Rosenbaum et al. demonstrated an increasing risk of vitamin B_12_ deficiency after the seventh post-operative year when ileal segments had been used for bladder augmentation. In their study, 6 out of 29 patients had low vitamin B_12_ levels (90). In a further study in which the ileocecal segment had been used, a slow decrease of serum vitamin B_12_ level was detected in more than 60% of patients; 8% underwent substitution after completion of a follow-up of 23 years (91).

In up to 35% of adult patients, substitution therapy is initiated following UD with ileal segments, such as ileal neobladder or ileocecal pouch (22, 74, 76, 77, 79). It is of note, however, that there are only single case reports of a total of five patients with clinical symptoms (one with neurological symptoms) seemingly related to vitamin B_12_ deficiency (72, 76, 79).

There are some controversies about the normal levels of vitamin B_12_ and the impact of age on requirements. A serum vitamin B_12_ level <100 ng/L is considered pathological, and some authors regard a range between 100 and 200 ng/L as borderline, whereas others recommend substitution of vitamin B_12_ if the serum level is below 200 ng/L (52, 64, 85, 92). It should be noted, that there is wide variation in vitamin B_12_ serum levels both in adults and children. In children, however, supplementation should be considered if serum levels drop below 200 ng/L, to be on the safe side (90, 91). Serum vitamin B_12_ levels should be checked for annually starting at year five following UD.

With regard to the substitution of vitamin B_12_ in patients with cobalamin deficiency, a randomized study provided evidence that oral therapy (2 mg per day) is as effective as parenteral application (1 mg intramuscularly) (57).

## Bowel Dysfunction

Chologenic diarrhea due to the loss of bile acids via the large bowel is one potential source of bowel dysfunction following UD using bowel segments. The pool of bile acids amount to around 2–4 g and circulates 5–10 times per day (referred to as “enterohepatic circulation”) (93). In the ileum, active reabsorption of conjugated bile acids involves a Na^+^-coupled co-transport system (94), and most of the conjugated bile acids are absorbed in the ileum (95). However, alongside active transport, the enterohepatic circulation includes also passive absorption of deconjugated bile acids from the jejunum and ileum. Under the influence of bacterial enzymes in the colon, deconjugation, 7α-dehydroxylation, and dehydrogenation of the conjugated bile acids occur (96) and only 0.2–0.4 g are lost through fecal excretion. This amount is normally synthesized by the liver. Therefore, the pool of bile acids remains by and large constant over time (97).

Resection of longer ileal segments may entail malabsorption of bile acids with subsequent excess transition of bile acids, water, and sodium into the colon. This can result in chologenic (bile acid) diarrhea (98). Multiple mechanisms of bile acid diarrhea are known (96, 99–102), but it remains still unclear which is the most important one (103). An increase of hepatic bile acid synthesis compensates for the loss. However, with increasing length of ileal resection, depletion of the bile acid pool can occur, resulting in malabsorption of fatty acids, which in turn can cause steatorrhea (104, 105). If the ileocecal valve is resected during UD, colonic organisms (e.g., Bacteroides ssp.) can enter the ileum, which is usually free of bacterial colonization. These microorganisms cleave bile acids from their conjungates. Free bile acids emulsify fat to a very low extent. As a consequence, micellar formation is reduced resulting in decreased fat absorption. This is a further cause of steatorrhea (106–108). Moreover, exclusion of the ileocecal valve decreases the intestinal transit time (0.8–2.5 h) (109) and thus, may increase stool frequency. Reconstruction of the ileocecal valve was previously recommended in patients with risk factors for developing post-operative diarrhea (110). However, in a long-term study using matched pairs, no difference was found between patients with and those without reconstruction of the ileocecal valve (111).

In the literature, only few reports have focused on bowel dysfunction after UD. In patients having undergone ileal or colonic conduit diversion, an increase of stool frequency was reported in 4–33%, following bladder augmentation or substitution in 7–59%, and after continent cutaneous diversion in 3–23% (112–119). In this context, it is noteworthy, however, that stool incontinence has been reported as surprisingly common in epidemiological studies, with an estimated prevalence of up to 20% depending on age, gender, and population (120, 121).

In patients with chologenic diarrhea, the principle of treatment is a reduction of bile acids in the colon. It has been known for a long time that cholestyramine effectively binds bile acids and reduces stool frequency after ileal resection (122). Patients with a long-term use of this substance are at risk of interference of cholestyramine with the absorption of fat-soluble vitamins such as vit. A, D, and K (123, 124). Therefore, vitamin levels should be checked. In patients with status post ileocecal pouch, no changes of the above vitamins were observed (125). Patients suffering from steatorrhea due to UD should be recommended a low fat diet. In patients with more severe bile acid malabsorption, cholylsarcosine (a bile acid analog) can be used for replacement (126) and substitution of fat-soluble vitamins may become necessary. Interestingly, there are only two reports on complications due to the lack of fat-soluble vitamins after bowel resection in adults (127, 128), whereas there are no reports thus far in patients after UD (22, 76).

## Conclusion

Metabolic consequences and disturbances are quite common in patients after UD using intestinal segments. However, careful patient selection for various types of UD, prophylactic substitution therapy (alkali supplementation, vitamin B_12_), early intervention in case of overt clinical symptoms, and life-long follow up, can avert or successfully treat major clinical problems in the majority of cases.

## Conflict of Interest Statement

The authors declare that the research was conducted in the absence of any commercial or financial relationships that could be construed as a potential conflict of interest.
